# Virulence Traits and Drug Resistance of STEC Isolated from Layer Poultry and *Rattus* Species

**DOI:** 10.3390/microorganisms14050977

**Published:** 2026-04-27

**Authors:** Tsepo Ramatla, Jane Nkhebenyane, Kgaugelo E. Lekota, Mpho Tawana, Oriel Thekisoe, Ntelekwane G. Khasapane

**Affiliations:** 1 Centre for Applied Food Safety and Biotechnology, Department of Life Sciences, Central University of Technology, 1 Park Road, Bloemfontein 9300, South Africankhasapanee@cut.ac.za (N.G.K.); 2 Unit for Environmental Sciences and Management, North-West University, Potchefstroom 2520, South Africa; 3 College of Agriculture & Environmental Sciences, University of South Africa, Private Bag X6, Roodepoort 1710, South Africa

**Keywords:** shiga-toxin producing *E. coli*, O-serogroups, antibiotic resistance properties, ESBL-producing STEC, *Ruttus* spp., chicken

## Abstract

Shiga toxin-producing *Escherichia coli* (STEC) has emerged globally as a critical enteric foodborne zoonotic pathogen with significant public health implications. This study aimed to isolate and characterize STEC strains from *Rattus* spp. and layer chickens, specifically evaluating their antimicrobial resistance (AMR) profiles and the prevalence of extended-spectrum β-lactamase (ESBL)-producing isolates. A total of 274 fecal samples were collected from *Rattus* spp. (n = 154) and layer chickens (n = 120). Isolates were characterized using standard microbiological techniques, PCR amplification of specific genes (including *uidA* and *stx*), and antimicrobial susceptibility testing via the disk diffusion method. Results: Of the 248 presumptive *E. coli* isolates, 237 (95.5%) were confirmed via *uidA* gene amplification. Fifty-eight isolates were confirmed as STEC, including key O-serogroups (O103, O111, O26, and O157). Resistance was most prevalent against colistin (39.6%) and streptomycin (20.6%), with 8.6% of isolates exhibiting multidrug resistance (MDR). Additionally, 19 isolates showed ESBL-producing phenotypes, and resistance genes for colistin, phenicols, aminoglycosides, and carbapenems were detected. The presence of STEC and MDR strains in both rodents and poultry highlights a high pathogenic potential and a serious zoonotic risk to public health, necessitating enhanced surveillance.

## 1. Introduction

*Escherichia coli* is a Gram-negative bacterium belonging to the Enterobacteriaceae family, which is commonly found in the gastrointestinal tracts of diverse animal species, including humans, where it typically resides as a commensal organism. *Escherichia coli* exhibits versatility as an opportunistic pathogen, encompassing multiple pathotypes including enterotoxigenic *E. coli* (ETEC), enteropathogenic *E. coli* (EPEC), extraintestinal pathogenic *E. coli* (ExPEC), and Shiga toxin-producing *E. coli* (STEC) strains, each capable of causing distinct diseases in humans [1,2,3]. Animals, particularly livestock and wildlife, frequently carry and serve as reservoirs for STEC strains, harboring virulent factors that can be transmitted to humans through direct contact or contaminated food and water [4,5,6]. Poultry is recognized as a significant reservoir for this *E. coli* pathotypes, which then increases the likelihood of zoonotic transmission, posing a considerable risk to human health through potential contamination of food products and environmental exposure [7].

Besides *E. coli* being detected in various studies, rodents have been conclusively identified as carriers of *E. coli*, highlighting their potential role in the transmission and dissemination of this bacterium [8,9,10]. Rodents serve as significant reservoirs for zoonotic pathogens, posing a substantial public health risk through the maintenance and transmission of infectious diseases [11,12,13].

Shiga toxin-producing *Escherichia coli* (STEC) is a subset of the highly pathogenic enterohemorrhagic *E. coli* (EHEC), a well-known classification of *E. coli* linked to severe foodborne diseases [5,14]. STEC strains are known for producing one or both types of Shiga toxins—*Stx1* and *Stx2* [15,16]—which bind specifically to the receptor globotriaosylceramide (Gb3), triggering a cascade of cellular events that disrupt protein synthesis, ultimately leading to the death of epithelial and endothelial cells [17]. STEC has been detected in rodents across various countries, including Argentina [17,18], Canada [15], and Brazil [19]. Research identifies rodents as key reservoirs of AMR *E. coli*, with resistance profiles distinct from livestock. This unique environmental signature underscores the role of both urban and rural rodents in the global maintenance of antimicrobial resistance [9,12,15].

Judicious antimicrobial selection informed by susceptibility testing is essential to optimize treatment success and curb the global trajectory of AMR. The rise of multidrug-resistant (MDR) *E. coli* is a major public health threat. Of particular concern is the increasing resistance to fluoroquinolones, beta-lactams, and last-resort antibiotics such as aminoglycosides, carbapenems, and colistin [20,21,22]. Among Enterobacterales, the most clinically significant antibiotic resistance genes include Beta-lactamase genes (*bla* genes), colistin resistance genes, tetracycline resistance genes, and aminoglycoside resistance genes [23,24].

A global escalation of ESBL-producing pathogens has been documented, exhibiting heterogeneous distribution patterns across nations [25]. The detection of ESBL-producing *E. coli* in humans, animals, and environmental sources constitutes a significant public health risk [26]. Empirical treatment strategies for ESBL-producing Enterobacteriaceae infections are facing significant obstacles due to rising antimicrobial resistance [26,27].

In South Africa, previous research has identified STEC across various animal reservoirs, including raw beef [28] and small ruminants such as sheep and goats [29,30]. The epidemiology and prevalence of STEC strains in rodents and layer chickens remain largely unknown. Applying a “One Health” perspective, this study uniquely evaluates the co-occurrence of STEC in both *Rattus* spp. and layer chickens within the same farm environments. We aim to characterize these STEC strains and compare their ESBL-producing phenotypes, AMR patterns, and underlying resistance genotypes to identify potential cross-species transmission risks.

## 2. Materials and Methods

### 2.1. Ethics Approval

Prior to the study’s commencement, the research proposal was approved by the North-West University Research Ethics Regulatory Committee (NWU-RERC), in accordance with the guidelines of the Animal Research Ethics Committee (NWU-00274-18A5). All methods in this study were approved by the scientific committee of the Unit for Environmental Sciences and Management of NWU. All experimental procedures were conducted by qualified personnel in strict adherence to the university’s ethical standards and regulations.

### 2.2. Sampling

Poultry farms in the Mafikeng area were identified using North West Department of Agriculture records, with representative sites randomly selected across four geographical quadrants. Following informed consent from farm owners, a stratified random sampling approach was implemented for layer chickens. This was supplemented by convenience sampling of *Rattus* spp., based on their observed presence and activity within the farm environments. In six commercial farms, a total of 274 fecal samples were collected from chickens (n = 120) and rats (n = 154). A total of 154 rats were collected from six commercial layer farms, and the target number of rodents (rats) was 150–200. Surfaces were cleaned with 70% ethanol before sampling. Rat cecum samples were harvested using a surgical blade and forceps. Chicken fecal samples were collected weekly from 3 floors per farm for representative distribution. Samples were packed in sterile bags, transported in an icebox, and processed immediately. Unanalyzed samples were refrigerated at −20 °C.

### 2.3. Rodent Identification

A total of 154 rats were captured using Sherman rat traps that were baited with a mixture of peanut butter and cheese. These rats were collected from chicken farms and, upon arrival at the lab, they were euthanized with 200 mg/kg of sodium pentobarbital [31]. The QIAamp DNA Blood and Tissue Kit (Qiagen, Hilden, Germany) was used to obtain DNA from skeletal muscle. Briefly, 20–24 mg tissue was lysed with proteinase K, incubated at 56 °C, and purified using spin columns. DNA was eluted in 200 μL Buffer AE and stored at −70 °C. The purity of DNA was determined spectrophotometrically from the ratio of absorbance at 260 and 280 nm (A260/A280). A ratio of between 1.7 and 2 indicates excellent DNA.

### 2.4. Molecular Identification of Rats

*Cytochrome oxidase 1* (*CO1*) genes were targeted for species identification. The final reaction mixture for both gene reactions was 25 μL and consisted of two μL of template DNA, 8.5 μL double distilled water, 2X Dream Taq Green PCR Master Mix (2X Dream Taq Green buffer, 4 mM MgCl_2_, 0.4 mM of each dNTP and 1 unit/μL of thermo stable Taq polymerase (Thermo Scientific, Johannesburg, South Africa), the primer mix contained 1 μM of each oligonucleotide primer. Positive PCR products were sent to Inqaba Biotechnical Industries (Pty) Ltd. in Pretoria, South Africa, for sequencing. Negative control was included by replacing the test DNA with 5 µL of sterile nuclease-free water. The cycling conditions and primer information are detailed in Supplementary Table S1.

### 2.5. Isolation of Escherichia coli

Fecal samples were then amassed from the caecum for further analysis. Furthermore, 120-layer chicken fecal samples were also collected within layer chickens’ houses of the same farms. A volume of 45 mL of peptone enrichment broth was added to the five grams of fecal samples and then incubated at 37 °C for 24 h. The enriched broth was then inoculated onto MacConkey agar (MAC) using the spread plate method. Generated colonies were examined for characteristic features. The *E. coli* colonies on MAC typically appeared as non-mucoid, pink or red colonies. For identity confirmation, about 2–3 presumptive pink to red (lactose-fermenting) colonies from each incubated plate were subcultured onto MAC. Pure isolates were preserved in 20% glycerol (Merck, Johannesburg, South Africa) and stored at −80 °C for further analysis.

### 2.6. Genomic DNA Extraction of E. coli

Genomic DNA was extracted from the isolates using the Fungal/Bacterial Soil Microbe DNA Mini Prep kit (Zymo-Research, Tustin, CA, USA), following the manufacturer’s protocol. The extracted DNA was eluted in 100 µL of DNA elution buffer into a clean 1.5 mL microcentrifuge tube and stored at −20 °C for subsequent molecular confirmation of *E. coli*, as well as detection of virulence and antibiotic-resistant genes.

### 2.7. Identification of Escherichia coli Using uidA PCR Assay

The PCR assay using the primers uidA-F (AAA ACG GCA AGA AAA AGC AG) and uidA-R (ACG CGT GGT TAC AGT CTT GCG) was utilized to amplify the *E. coli uidA* housekeeping gene, as described by Ramatla et al. [32]. The PCR assay was conducted on a ProFlex PCR System (Applied Biosystems, Waltham, MA, USA) with a total reaction mixture of 25 μL containing 12.5 μL AmpliTaq Gold DNA Polymerase Master Mix (Applied Biosystems, Carlsbad, CA, USA), 2 μL of DNA template, 1 μL each of 10 μM forward and reverse primer, and 8.5 μL of nuclease-free PCR water. PCR cycling conditions included: 95 °C for 5 min for initial denaturation, and then 40 cycles of 95 °C for 40 s of denaturation, 60 °C for 30 s of annealing, and 72 °C for 40 s of elongation, and 72 °C for 7 min of final extension. Negative control was included using nuclease-free water as a DNA-free template. For standardization, 100 bp and 1 kb DNA molecular weight marker (PROMEGA, Madison, WI, USA) were used to determine PCR amplicon size. A total of 5 µL of the PCR amplicons were electrophoresed on a 1.5% agarose gel for 45 min at 80 V and stained with ethidium bromide for visualization under UV light.

### 2.8. Identification of E. coli Using the 16S rRNA Gene

The *E. coli* isolate that tested positive for the *uidA* PCR assay underwent additional screening via *16S rRNA* PCR, utilizing the primer pair 27F (AGA GTT TGA TCM TGG CTC AG) and 1492R (GGT TAC CTT GTT ACG ACT T) [33]. For PCR assays targeting *16S rRNA* gene, a 25 µL reaction mixture was prepared, comprising 12.5 µL of 2X AmpliTaq Gold DNA Polymerase, 10 µM of each primer, 2 µL of template DNA, and 8.5 µL of nuclease-free water. Optimized PCR conditions included an initial 4 min denaturation at 96 °C, followed by 30 cycles of denaturation (94 °C, 30 s), annealing (57 °C, 30 s), and extension (72 °C, 1 min), and a final 10 min extension at 72 °C. The resulting PCR products were purified using ExoSAP-IT, sequenced using the BigDye Terminator v3.1 kit (ThermoScientific, Waltham, MA, USA), and analyzed on the SeqStudio genetic analyzer at North-West University, Potchefstroom, South Africa.

### 2.9. Detection of Serogroups and Virulence Genes Using PCR

In this study, we utilized previously published primers to detect virulent genes (*eaeA*, *stx1* and *stx2*) using PCR. Individual PCR reactions for each virulence gene were set up in a 25 µL volume, comprising 12.5 µL of AmpliTaq Gold 360 PCR Master Mix (Applied Biosystems, Carlsbad, CA, USA). This master mix contained AmpliTaq Gold DNA Polymerase (0.05 units/µL), Gold buffer (930 mM Tris/HCl, pH 8.05), 100 mM KCl, 400 mM of each dNTP, and 5 mM MgCl_2_. The reaction mixture was supplemented with 2.5 mM of each primer, 2 µL of template DNA, and ddH_2_O to reach the final volume. PCR cycling conditions included: 95 °C for 5 min for initial denaturation, and then 40 cycles of 95 °C for 40 s of denaturation, 55–50 °C for 30 s of annealing, and 72 °C for 40 s of elongation, and 72 °C for 7 min of final extension (Supplementary Table S1). Negative control was included by replacing the test DNA with 5 µL of sterile nuclease-free water. The cycling conditions and primer information are detailed in Supplementary Table S1.

### 2.10. Antimicrobial Susceptibility Testing

Pure *E. coli* isolates were subjected to antibiotic susceptibility testing utilizing the Kirby–Bauer disc diffusion method on Müller–Hinton (MH) agar plates at a depth of 4–6 mm, as per standard protocol. Pure cultures of isolates from layers and *Rattus* spp. were used to determine antibiotic resistance patterns. The antibiotic discs used in this study included aminoglycosides (streptomycin, 10 μg; gentamicin, 10 μg), phenicol (chloramphenicol, 30 μg), fluoroquinolones (ciprofloxacin, 5 μg), nalidixic acid (30 μg), and carbapenems (meropenem, 10 μg; imipenem, 10 μg). These discs were used to test for antibiotic resistance in *Enterobacteriaceae* isolates, following the Clinical and Laboratory Standards Institute (CLSI) antimicrobial susceptibility testing standards [34]. Colistin susceptibility was determined using the broth microdilution method, as recommended by the Clinical and Laboratory Standards Institute [35]. Antibiotic discs were obtained from ThermoFisher Scientific (Thermo Fisher, Johannesburg, South Africa). *Escherichia coli* ATCC 25922 served as a quality control reference strain for antimicrobial susceptibility testing. Isolates were classified as multidrug-resistant (MDR) if they exhibited resistance to three or more classes of antibiotics.

### 2.11. Screening of ESBL-Producing E. coli

Phenotypic confirmation of ESBL-producing *E. coli* was performed using CHROMagar ESBL (CHROMagar, Saint-Denis, France). This medium differentiates ESBL-producing *E. coil*, which appears as red colonies. The selection of these isolates was based on their ability to produce extended-spectrum *β*-lactamases (ESBL). Reference strains used in this study included *E. coli* ATCC 10536, a pathogenic *E. coli* strain, and ESBL-producing *E. coli* ATCC 35218 (Microbiologics, St Cloud, MN, USA) [36].

### 2.12. Molecular Detection of Resistance Genes

The *E. coli* isolates were screened for the presence of *β*-lactamase resistance encoding genes, including *bla*_SHV_, *bla*_OXA_, *bla*_CARB_, *bla*_TEM_, *bla*_CTX-M_, and specific *bla*_CTX-M_ groups (*bla*_CTX-M-1_, *bla*_CTX-M-2_, *bla*_CTX-M-8_, *bla*_CTX-M-9_, *bla*_CTX-M-15_, and *bla*_CTX-M-25_). The genomic DNA extracted from *E. coli* isolates was screened for the presence of colistin genes (*mcr-1*, *mcr-2*, *mcr-3*, *mcr-4* and *mcr-5*), carbapenems (*bla*_KPC_ and *bla*_VIM_), phenicol (*catI*, *catII*, *catIII*, *catIV* and *floR*), aminoglycoside [*strA*, *strB*, *aadA*, *aac(6′)-Ib*, *armA*, *rmtB* and *aadE*], and fluoroquinolones (*qnrA*, *qnrD*, *qnrS* and *aac(6′)-Ib-cr*). PCR assays were performed using previously described primers (Supplementary Table S2). The PCR reaction mixture consisted of 2 μL template DNA, 8.5 μL nuclease-free water, 1 μL each of the oligonucleotide primers, and 12.5 μL 2X DreamTaq Green Master Mix (Thermo Fisher Scientific, Johannesburg, South Africa), containing 0.4 mM dNTPs, 4 mM MgCl_2_, and a loading buffer. The amplification program included an initial denaturation step at 94 °C for 5 min, followed by 35 cycles of amplification, comprising 1 min denaturation at 94 °C, 1 min (annealing temperature range from 46 to 66 °C) and 1 min primer extension at 72 °C. A final 10 min extension step at 72 °C completed the program.

### 2.13. Data Analysis

Statistical analysis was performed using Microsoft Excel 2016 (Microsoft Corporation, Redmond, WA, USA) and IBM SPSS Statistics version 26 (IBM Corporation, Armonk, NY, USA). The sequenced *16S rRNA* gene of the representative isolates were aligned with BLASTn (version: 2.17.0 http://www.ncbi.nlm.nih.gov/BLAST/, accessed on 13 September 2025) for identity match on the database of National Center for Biotechnology Information database (NCBI). The ChipPlot (https://www.chiplot.online/#, accessed on 13 September 2025) was utilized to generate heatmap plots of the virulence and antibiotic resistance profiles of the *E. coli* bacteria isolated in this study. Furthermore, to compare whether the results were statistically significant or not based on the phenotypic and genotypic AMR and virulence genes, Fisher’s test was conducted. A *p*-value of less than 0.05 was regarded as significant.

## 3. Results

### 3.1. Rattus *spp.* Identification

Identification of rodent species was performed based on the analysis of the *cytochrome oxidase 1* (*CO1*) gene sequence, obtained as described in the materials and methods section. Out of the 154 rodents screened, species identification via *CO1* sequencing confirmed 64.3% (n = 99) as *Rattus rattus* and 35.7% (n = 55) as *Rattus tanezumi* through comparison with NCBI GenBank records. The obtained *CO1* sequences were deposited in GenBank with accession numbers MK645246-MK645295.

### 3.2. Identification of E. coli Strains from Rattus *spp.* and Layer Chickens’ Feces

A total of 248 presumptive *E. coli* isolates were subjected to further analysis. A total of 237 *E. coli* isolates were confirmed with the PCR amplification of the *uidA* gene, a housekeeping gene characteristic of *E. coli*, with 94 isolates from layer chickens and 143 from *Rattus* spp. detected as *E. coli*. The *E. coli* isolates were found in the six farms screened: Farm E had the highest number with 66 (27.8%), followed by Farm A with 52 (21.9%), Farm D with 41 (17.2%), Farm C with 23 (9.7%), and lastly Farm B with 20 (8.4%) (Table 1).

The 16S rRNA from two isolates from rodents and another two from chickens were sequenced to corroborate that they were *E. coli* isolates. The BLASTn results confirmed the identity of the *E. coli* isolates, showing high nucleotide similarities (98.2–99.5%) to reference *E. coli* strains from the GenBank database. The obtained sequences were deposited in GenBank under accession numbers PV951861 and PV951862 (*Rattus* spp.), and PV952222 and PV952223 (Layer chickens).

### 3.3. Detection of STEC Strains and Serogroup Assignation

**Figure 1:**
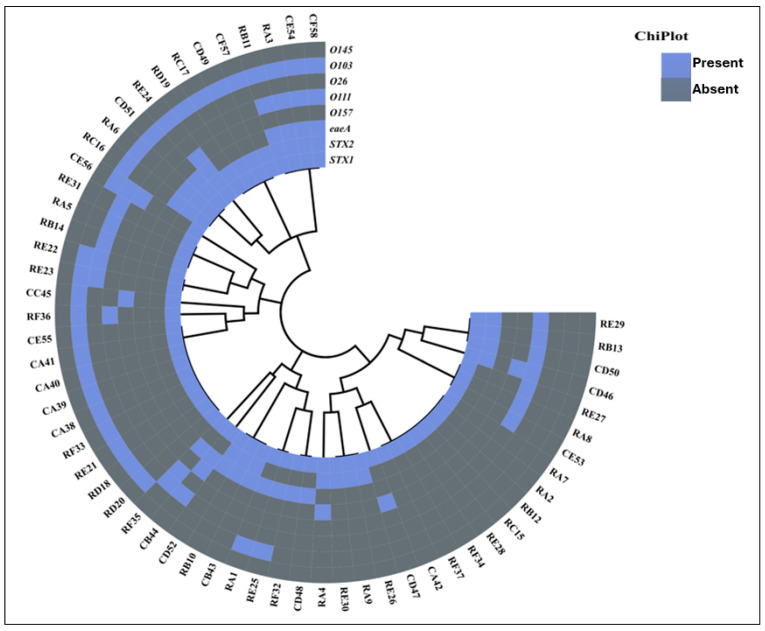
Heatmap showing O-serogroup and STEC genes detected in 58 STEC isolates from the feces of *Rattus* spp. and layer chickens. Light blue and dark blue indicate absence and presence of O-serogroup and STEC genes, respectively. Isolate names: R = *Rattus* spp., C = chickens. https://www.chiplot.online/# (accessed on 26 November 2025).

STEC strains were identified via PCR amplification of specific virulence markers. Among the confirmed *E. coli* isolates, the *stx1* gene was the most prevalent at 24.4%. Within these positive isolates, 9.7% harbored the *stx2* gene, while the *eaeA* gene was detected in 17.2%. A total of 17.2% isolates carried a mix of all three genes; namely, *stx1*, *stx2*, and *eaeA*. Furthermore, 20.6% of isolates carried both *stx1* and *stx2* genes, while 10.3% isolates harbored a mix of both *stx1* and *eaeA* genes (Figure 1). Molecular characterization confirmed 58 *E. coli* isolates as STEC.

The 58 confirmed STEC isolates were further subjected to O-serogrouping. The O103 O-serogroup was the most detected, representing 48.2% of all STEC isolates. This was followed by O111 with 14 (24.1%), O26 with 13.8%, then O157 with 10.3%. Notably, the O145 O-serogroup was not detected in this study (Figure 1).

### 3.4. Phenotypic Antibiotic Resistance and ESBL-Producing STEC Isolates

**Figure 2:**
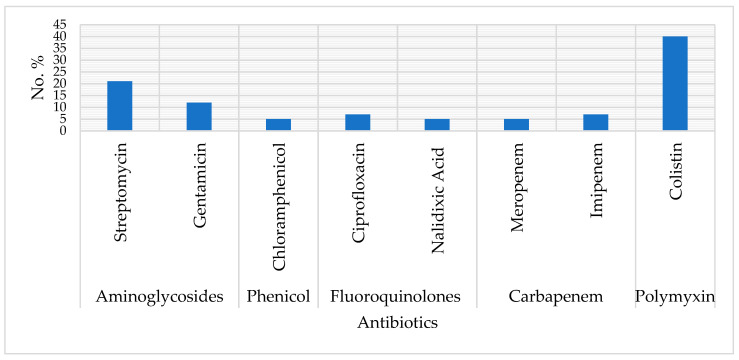
The antibiotic resistance profiles from the STEC isolated from Layer Chickens and *Rattus* species.

The STEC isolates exhibited resistance to the tested antibiotics depending on the isolate: 39.6% were colistin resistant (MIC ≥ 4 µg/mL), 20.6% showed resistance to streptomycin, 12.0% to gentamicin, 6.8% to ciprofloxacin, 6.8% to imipenem and 5.1% to chloramphenicol, nalidixic acid or meropenem (Figure 2). A total of 8.6% of the isolates were multidrug resistant, showing resistance to three or more antimicrobial classes.

### 3.5. Antimicrobial Resistance Genes for STEC E. coli Isolates

The distribution of extended-spectrum *β*-lactamase genes among the isolates was as follows: *bla*_CTX-M_ (32.8%), *ampC* (31.0%), *bla*_CTX-M-1_ (10.3%), *bla*_CTX-M-15_ (5.2%) and *bla*_CTX-M-8_ (3.4%). Multiple *β*-lactamase genes co-existed in 8.6% isolates. The most prevalent combination consisted of two genes, specifically *bla*_CTX-M_ and *bla*_CTX-M-1_, which occurred in four isolates. Furthermore, one notable isolate was found to co-harbor both *bla*_CTX-M_ and *bla*_CTX-M-8_.

Among the screened carbapenem resistance genes, the *bla*_KPC_ resistance gene was found more frequently, in 17.2% of the isolates, while *bla*_VIM_ appeared in 5.1% of them.

The prevalence of mobile colistin genes among the isolates was as follows: *mcr-1* (69%), *mcr-2* (31%), and *mcr-4* (40%). Three isolates carried the *mcr-1*, *mcr-2*, and *mcr-4* genes together, while others had different combinations of these genes. This included three isolates with *mcr-1* and *mcr-2*, six isolates with *mcr-1* and *mcr-4*. The *mcr-3* and *mcr-5* were not detected in this study.

For aminoglycoside, 12.0% harbored *strA*, 6.8% carried *aadA*, and only 5.1% carried *aadE*. Furthermore, chloramphenicol resistance genes were detected, with *catI* present in 10.3% and *catIII* present in 5.1% of the isolates. The *catII*, *catIV*, *strB*, *armA*, *rmtB* and *aac(6′)-Ib* genes were not detected.

### 3.6. The Phenotype and Genotype ESBL-Producing STEC Isolates

Considering only the 19 isolates found to be positive ESBL producers through the CHROMEagar tests, the following *bla*_CTX_ genes were detected: *bla*_CTX-M_ (47.4%), *bla*_CTX-M-1_ (21.1%), *bla*_CTX-M-8_ (10.5%), and *bla*_CTX-M-15_ (10.5%) (Table 1).

### 3.7. Coexistence of Phenotypic and Genotypic Antibiotic Resistance

The resistance gene profiling of the *E. coli* isolates revealed several important findings. The *catI* gene, which was detected in four chloramphenicol-resistant isolates, was associated with resistance to chloramphenicol. Carbapenem resistance was associated with the *bla*_KPC_ gene, identified in three isolates resistant to meropenem and four isolates resistant to imipenem. This included two isolates resistant to both meropenem and imipenem. Additionally, colistin resistance was connected to various *mcr* genes. Three isolates had combinations of *mcr-1* and *mcr-2*, six isolates with *mcr-1* and *mcr-4*, four isolates with only *mcr-1*, and one isolate with *mcr-4*.

### 3.8. Statistical Analysis Results

Chi-square tests showed no significant associations (*p* > 0.05) between AMR genotypes/phenotypes, *stx* genes, ESBL genes, or specific serogroups (*eaeA*, O111, O26, O103, and O145). However, strong correlations were found between *catI* and *bla*_KPC_ genes and phenotypic chloramphenicol resistance (i.e., *p* < 0.015 and *p* < 0.029 in rats, respectively), and between *aadA* and streptomycin resistance (*p* < 0.029).

## 4. Discussion

This study reveals an alarmingly high prevalence of *E. coli* in layer chickens and rodents, accompanied by widespread antimicrobial resistance and arrays of antibiotic-resistant genes, highlighting a pressing public health concern. Notably, chickens and *Rattus* spp. act as natural reservoirs, harboring *E. coli* in their gastrointestinal tracts [32,37]. To detect and confirm the presence of *E. coli*, we utilized PCR assay, a widely adopted method for identifying pathogens in food, animal, clinical, and environmental samples. Specifically, the *uidA* gene served as a confirmatory marker, enabling identification of the *E. coli* isolates. The findings of this study revealed that 237 isolates were identified as *E. coli*.

Among the 237 *E. coli* isolates identified, 58 (24.4%) were classified as Shiga toxin-producing *E. coli* (STEC). The prevalence of virulence genes revealed that *stx1* was detected in 100% of STEC strains, while *stx2* was present in 39.6%. Additionally, the *eaeA* gene associated with enteropathogenic *E. coli* (EPEC), was found in 29.3% of the isolates. This is contrary to previous studies in South Africa, where *stx2* was most prevalent in sheep and goats [36]. In contrast, a study in Ghana reported distinct findings, where none of the *E. coli* isolates harbored *stx1*, *stx2*, or *eaeA* genes [38]. These variations likely reflect differences in sample sources, sampling methodologies, geographic locations, and host populations, highlighting the complexity and diversity of *E. coli* strains. *E. coli* strains possessing the *stx1* gene are linked to the risk of inducing severe diarrhea, especially in immunocompromised individuals [39]. This highlights the necessity for further characterization of *stx1* subtypes in livestock, including their geographic distribution, and implications for both animal and human health. Furthermore, investigating *stx1* subtypes in chickens and rodents is crucial for understanding their genetic diversity and pathogenic potential. The detection of EPEC in chicken highlights its zoonotic potential, suggesting a potential risk of gastrointestinal illnesses in humans who handle or consume contaminated poultry products [2].

All STEC isolates from chickens and rodents in this study were successfully serotyped using PCR. Notably, none of the 58 *stx*-positive samples belonged to the O145 serogroup. Only 2.5% of the STEC isolates harbored O157. The relatively low prevalence of O157 STEC observed in this study indicates a reduced risk of O157 infection transmission to humans from these animals. In the present study, we detected only three non-O157 STEC serogroups (O111, O26, and O103) in chickens and rodents’ feces. Notably, STEC O103 strains were identified in a significant proportion of samples in this study, which have previously been implicated in numerous foodborne illness outbreaks in England and Wales [40].

Antimicrobials play a crucial role in managing microbial infections in both veterinary and human medicine. Additionally, they are often used as growth promoters in livestock production [32,41]. The rise of antimicrobial resistance is a global concern, prompting the WHO to prioritize ESBL-producing Enterobacteriaceae as a critical threat requiring new treatments [42,43]. Among Enterobacterales, the most critical antibiotic resistance genes include Beta-lactamase genes (*bla* genes), colistin resistance genes (*mcr* genes), tetracycline resistance genes (*tet* genes), and aminoglycoside resistance genes, which confer resistance to various clinically important antibiotics [44,45]. Antibiotic susceptibility testing revealed that 39.6% STEC isolates were resistant to colistin, which was significantly lower (81.5%) in Chile from cattle and swine [46].

In South Africa, antibiotics such as ampicillin, quinolones, streptomycin, and florfenicol have been widely used in animal husbandry for decades to prevent and treat animal diseases, as well as to promote growth [47]. This extensive use of antibiotics may contribute to the high resistance rates (ranging from 5.1% to 39.6%) observed among STEC isolates to these drugs, along with the notable multidrug resistance (MDR) rate of 8.6% identified in this study. Antibiotic susceptibility testing showed that 39.6% of STEC isolates were resistant to colistin, which was significantly lower (81.5%) in Chile [46].

The persistence of colistin-resistant genes in STEC represents a serious threat to global food safety and public health, with poultry farming being a key area of concern. In this study, 39.6% of STEC isolates exhibited resistance to colistin. The findings are slightly lower than those reported in a Chilean study, which found that 81.5% of STEC isolates derived from cattle and swine exhibited resistance to colistin [46]. In this study, three colistin resistance genes were detected: *mcr-1* (69%), *mcr-2* (31%), and *mcr-4* (40%). Similar findings have been documented in Europe; Garcia et al. [48] identified *mcr-1* and *mcr-4* in Spain, while Ewers et al. [49] reported *mcr-1* and *mcr-2* in German swine.

The high prevalence of colistin resistance (39.6%) observed in this study suggests a well-established environmental resistome maintained by both layer chickens and commensal rodents. Historically, the widespread use of colistin in poultry for growth promotion and group therapy created a powerful selection pressure that favored the persistence of resistant strains [50]. The detection of plasmid-mediated genes is particularly significant, as these mobile genetic elements allow resistance to persist and spread horizontally even in the absence of active antibiotic use [51]. The transmission dynamics between *Rattus* spp. and poultry are likely driven by a fecal–oral cycle within the farm’s shared agricultural niche. Rodents act as biological bridges, contaminating feed and water systems with infected feces while moving between manure pits and clean production areas [52]. This cycle of cross-species transmission underscores the critical role of rodents as overlooked reservoirs that maintain last resort antibiotic resistance in livestock environments, posing a significant challenge to farm biosecurity [53].

The colistin resistance was linked to various *mcr* genes, including *mcr-1*, *mcr-2*, and *mcr-4*, often found in combination among the isolates. The diverse genetic mechanisms of IncI and IncHI2/HI2A plasmids carrying *mcr-1*, such as the ISApl1 insertion element, facilitate the high rate of horizontal transfer of *mcr-1*, enabling its rapid spread [54]. Polymyxins like colistin are classified as critically important antimicrobials. However, their effectiveness is declining due to overuse and misuse in livestock production, compromising their ability to treat life-threatening infections [54,55]. Resistance rates to other antibiotics were also observed, including Streptomycin (20.6%), Gentamicin (12.0%), Ciprofloxacin (6.8%), and Imipenem (6.8%), which aligns with findings from previous studies [2,54,56]. 

Beta-lactamase (*bla*) genes are among the most prevalent antibiotic resistance determinants in Enterobacteriaceae and other bacterial species, conferring resistance to beta-lactam antibiotics [57,58]. Specifically, the *bla*_TEM_ genes are known to mediate resistance to *β*-lactam antibiotics such as ampicillin [59]. Our study revealed a notably high prevalence (32.7%) of ESBL-producing STEC in broilers and rodents, consistent with findings from similar studies conducted in chicken production facilities in Bangladesh, India and Ghana [2,60,61]. In this study, none of the ESBL-producing STEC isolates carried *bla*_TEM_ genes. However, four *bla*_CTX-M_ genes were detected: *bla*_CTX-M_ (32.8%), *bla*_CTX-M-1_ (10.3%), *bla*_CTX-M-8_ (3.4%), and *bla*_CTX-M-15_ (5.2%). Notably, co-existence of these genes was observed in 8.6% of the STEC isolates.

The co-existence of these genes was found in 8.6% STEC isolates. These results are consistent with previous studies from Saudi Arabia (73.9%), Germany (69.9%), and Nigeria (44.7%), which also reported high occurrences of *bla*_CTX-M_ [62,63,64]. Among the *bla*_CTX-M_ groups, *bla*_CTX-M-1_ exhibited the highest detection rate, at 21.1%. Our findings are supported by previous studies, including a German investigation that reported a 6.9% prevalence of *bla*_CTX-M-1_ in *E. coli* isolates from humans, cattle, and poultry in Pakistan [65], and a Saudi Arabian study that found a higher prevalence of 54.3% in fecal specimens from animals [53]. Globally, *bla*_CTX-M-15_ and *bla*_CTX-M-14_ are the most commonly reported *bla*_CTX-M_ enzymes, widespread across human, animal, and environmental niches [66,67]. Studies have revealed a link between the dissemination of *bla*_CTX-M_-producing enzymes and *E. coli* strains belonging to the ST131 clonal group. Notably, ST131 isolates have been found to harbor multiple *bla*_CTX-M_ types, including the predominant *bla*_CTX-M-15_ [26]. ESBL-producing Enterobacteriaceae infections are becoming increasingly hard to treat empirically [27]. Notably, ESBL-producing genes were detected in STEC isolates from both layer chickens and rodents, with a significantly higher prevalence of STEC isolates found in rodents compared to layers.

Among the carbapenem-resistance genes identified, *bla*_KPC_ emerged as the most prevalent, detected in 17.2% of isolates, followed by *bla*_VIM_, found in 5.1%. Carbapenems, often considered the last line of defense against ESBL-producing *Enterobacteriaceae* are increasingly compromised by rising resistance, narrowing treatment options and complicating infection control efforts in healthcare settings worldwide [68]. An additional noteworthy finding was the detection of aminoglycoside-resistance genes, which exhibited varying frequencies. Notably, genes such as *strA*, *aadA*, and *aadE* were detected, further illustrating the multifaceted nature of antimicrobial resistance in these isolates.

A concerning finding of our study is the high detection frequency of *mcr* genes in STEC isolates, predominantly from rodents. This raises alarming concerns, as rodents can thrive in various environments, including human surroundings, facilitating the spread and maintenance of resistance genes in uncontrollable settings. Colistin and carbapenems are critical antibiotics of last resort, used to combat MDR bacterial infections in humans [69,70]. Notably, this study marks the first detection of *mcr-1*, *mcr-2*, and *mcr-4* genes in STEC isolates from rodents and chickens in South Africa. Colistin usage has surged dramatically in recent years, particularly in countries where it has proven effective in treating antibiotic-resistant infections [36]. These findings highlight the need for enhanced surveillance, stricter antimicrobial stewardship, and targeted interventions to mitigate the risk of zoonotic transmission and preserve the efficacy of critical antibiotics.

The lack of a significant association between virulence determinants and antimicrobial resistance observed in this study [48,49] is a noteworthy finding that suggests these traits may be evolving independently within the STEC population. This lack of correlation has been previously documented in other European porcine studies. For instance, García et al. [48] found that while *mcr* genes were highly prevalent in Spanish farms, their distribution among different pathotypes did not show a linear relationship with specific virulence profiles, even within dominant clonal groups like ST10. Similarly, Ewers et al. [49] reported that while *mcr-1* and *mcr-2* were identified in various porcine *E. coli* lineages in Germany, these resistance genes were often carried on highly mobile IncX4 or IncI2 plasmids, which can move between strains regardless of their underlying virulome.

This finding is particularly alarming, as rodents are highly adaptable and capable of thriving in wide range of environments, including those closely associated with human activity. Consequently, they can serve as reservoirs and vectors for the dissemination of resistance genes, making control efforts increasingly challenging. The dynamic interactions between the environment, rodents, poultry, and humans create a complex network through which antibiotic-resistant bacteria and genes can circulate. This is further exacerbated by the use of antimicrobials in poultry farming, which contributes to the selection and persistence of resistant strains [70,71].

## 5. Conclusions

This study demonstrates a concerning convergence of virulence and AMR in Shiga toxin-producing Escherichia coli (STEC) isolated from layer chickens and Rattus spp., posing a significant threat to public health and food safety. Our findings identify rodents as critical reservoirs and vectors that facilitate the transmission of resistant STEC within poultry facilities and throughout the food chain, highlighting a major biosecurity gap. To mitigate this risk, it is imperative to implement integrated pest management strategies that prioritize rodent exclusion from feed and water systems, alongside intensified surveillance for last-resort resistance genes like mcr. Furthermore, the high prevalence of colistin resistance underscores the urgent need for stricter antimicrobial stewardship and the transition toward antibiotic alternatives in livestock production. Future research utilizing Whole Genome Sequencing (WGS) is essential to map the precise evolutionary pathways of these resistant clones and to evaluate the spill over risk to farm workers. Furthermore, to guarantee that emerging antibiotics, including newer agents like omadacycline, are explored in the veterinary setting, comprehensive pharmacovigilance studies assessing their real-world safety profiles are necessary [72]. Simultaneously, the investigation of non-conventional antimicrobial strategies, like food-derived bioactive peptides (FDBPs), presents a promising potential owing to their multitarget activity, favorable safety profiles, and capacity to penetrate biological barriers. Exploration of innovative drug delivery platforms, particularly those involving metal-ion mediation, could improve the stability, bioavailability, and targeted action of these biomolecules [73].

## Figures and Tables

**Table 1:** The *β*-lactamase-encoding genes patterns of the ESBL-producing *E. coli* isolates isolated from fecal samples of layer chickens and *Rattus* spp.

	Isolates	ESBL	*bla*_CTX-M_	*bla*_CTX-M-1_	*bla*_CTX-M-8_	*bla*_CTX-M-15_
1	RA1	+	+	+	−	−
2	RA3	+	−	−	−	−
3	RA4	+	−	−	−	−
4	RA6	+	+	−	+	−
5	RA9	+	+	−	−	−
6	RB12	+	−	−	−	−
7	RB13	+	+	+	−	−
8	RB14	+	−	−	−	−
9	RC17	+	−	−	−	−
10	RE21	+	−	−	−	−
11	RE27	+	−	−	−	−
12	RE28	+	−	−	+	−
13	CA40	+	−	−	−	−
14	CB43	+	+	−	−	−
15	CD48	+	+	−	−	−
16	CD49	+	+	+	−	−
17	CD50	+	+	−	−	−
18	CE56	+	+	+	−	−
19	CF58	+	−	−	−	+

+ = positive; − = negative.

## Data Availability

The data and materials supporting this study are available from the corresponding author upon reasonable request. The genomic sequences of the two strains analyzed have been deposited in the National Center for Biotechnology Information (NCBI) GenBank nucleotide sequence database, accessible through the National Library of Medicine, and assigned the accession number https://www.ncbi.nlm.nih.gov/nuccore/PV951861, https://www.ncbi.nlm.nih.gov/nuccore/PV951862, https://www.ncbi.nlm.nih.gov/nuccore/PV952222, https://www.ncbi.nlm.nih.gov/nuccore/PV952223 (all accessed on 20 April 2026).

## Supplementary Materials

The following supporting information can be downloaded at: https://www.mdpi.com/article/10.3390/microorganisms14050977/s1, Table S1: Primer pairs used in this study to determine the *E. coli* pathotypes and O-serogroups; Table S2: List of antibiotic resistance genes primers used in this study. References [14,70,74,75,76,77,78,79,80,81,82,83,84] are cited in the Supplementary Materials.
