# Supplementary material for: Virulence Traits and Drug Resistance of STEC Isolated from Layer Poultry and Rattus Species
Source: Microorganisms. 2026 Apr 27;14(5):977. doi: 10.3390/microorganisms14050977 (PMC13209377; doi:10.3390/microorganisms14050977)
Supplement: Supplementary file 1 [file microorganisms-14-00977-s001.zip › microorganisms-4219741-supplementary.pdf]

SUPPLEMENTARY MATERIALS

Virulence traits and antimicrobial resistance patterns of Shiga Toxin-Producing *Escherichia coli* from Layers and *Rattus* species

Tsepo Ramatla[1*], Jane Nkhebenyane[1], Kgaugelo E. Lekota[2], Mpho Tawana[3], Oriel Thekisoe[2], and George Khasapane[1]

[1]Centre for Applied Food Safety and Biotechnology, Department of Life Sciences, Central University of Technology, 1 Park Road, Bloemfontein, 9300, South Africa.

[2]Unit for Environmental Sciences and Management, North-West University, Potchefstroom 2520, South Africa.

[2] Department of Agriculture and Animal Health, University of South Africa, Florida, South Africa

*** Correspondence:**

Corresponding Author: Tsepo Ramatla, PhD

Tell: +27 51507 3911, Email: tramatla@cut.ac.za

Supplementary Table S1: Primer pairs used in this study to determine the *E. coli* pathotypes, O-serogroups and rodents.

Pathotype & O-serogroup	Primer	Sequence of primers (5–3′)	Amplification length (bp)	Annealing temp (°C)	Reference
**Pathotype**	EAE1	TCAATGCAGTTCCGTTATCAGTT	450	55	Omolajaiye et al., 2020
	EAE2	GTAAAGTCCGTTACCCCAACCTG			
	EVT1	CAACACTGGATGATCTCAG	349	55	Wolde et al., 2022
	EVT2	CCCCCTCAACTGCTAATA			
	EVS1	ATCAGTCGTCACTCACTGGT	110	55	Wolde et al., 2022
	EVS2	CTGCTGTCACAGTGACAAA			
**O-serogroup**	O145F	CCATCAACAGATTTAGGAGTG	609	58	Ranjbar et al., 2017
	O145R	TTTCTACCGCGAATCTATC			
	O103F	TTGGAGCGTTAACTGGACCT	321	58	Ranjbar et al., 2017
	O103R	GCTCCCGAGCACGTATAAG			
	O111F	TAGAGAAATTATCAAGTTAGTTCC	406	58	Ranjbar et al., 2017
	O111R	ATAGTTATGAACATCTTGTTTAGC			
	O26F	CAGAATGGTTATGCTACTGT	423	58	Ranjbar et al., 2017
	O26R	CTTACATTTGTTTTCGGCATC			
	O157F	CGGACATCCATGTGATATGG	259	58	Ranjbar et al., 2017
	O157R	TTGCCTATGTACAGCTAATCC			
**Rodents' identification**	BatL5310	CCTACTCRGCCATTT TACCTATG	762	60	Ramatla et al., 2019
	R6036R	ACTTCTGGGTGTCCAAAGAATCA			

Supplementary Table S2. List of antibiotic resistance genes primers used in this study.

Class	Target gene	Primer	Primer sequence (5´ → 3´)	Amplicon size (bp)	Annealing temp (°C)	References
**Colistin**	*mcr*-1	mcr-1-F<br>mcr-1-R	TATCGCTATGTGCTAAAGCCTG<br>CGTCTGCAGCCACTGGG	1139	56	Jousset et al., 2019
	mcr-2	mcr-2-F<br>mcr-2-R	TATCGCTATGTGCTAAAGCCTG<br>AAAATACTGCGTGGCAGGTAGC	816	56	Jousset et al., 2019
	mcr-3	mcr-3-F<br>mcr-3-R	CAATCGTTAGTTACACAATGATGAAG<br>AACACATCTAGCAGGCCCTC	676	56	Jousset et al., 2019
	mcr-4	mcr-4-F<br>mcr-4-R	ATCCTGCTGAAGCATTGATG<br>GCGCGCAGTTTCACC	405	56	Jousset et al., 2019
	mcr-5	mcr-5-F<br>mcr-5-R	GGTTGAGCGGCTATGAAC<br>GAATGTTGACGTCACTACGG	207	56	Jousset et al., 2019
**Fluoroquinolones**						
	*qnr*A	qnrA-F<br>qnrA-R	ATTTCTCACGCCAGGATTTG<br>GAGATTGGCATTGCTCCAGT	413		Xu et al., 2015
	*qnr*D	qnrD-F<br>qnrD-R	GCTGGAGCTTGTCAGGGATT<br>TGCTGCGAGATATCATGCGT	585		
	qnrS	qnrS-F<br>qnrS-R	CCCCATGCCCGAAGTTATCA<br>ACTGCTTGGAGTGTGTTGGT	457		Xu et al., 2015
	aac(6´)-Ib-cr	aac(6´)-Ib-cr-F<br>aac(6´)-Ib-cr-R	ATATGCGGATCCAATGAGCAACGCAAAAACAAAGTTAG<br>ATAGCGAATTCTTAGGCATCACTGCGTGTTCGCT	544	66	

**β-lactam**						
	*amp*C	AmpC -F<br>AmpC R	GTGACCAGATACTGGCCACA<br>TTACTGTAGCGCCTCGAGGA	822	61	Liu et a., 2018
	*bla*SHV	SHV-F<br>SHV-R	CACTCAAGGATGTATTGT G<br>TTAGCGTTGCCAGTGCTCG	885	55	Ramatla et al., 2022
	*bla*OXA	OXA-F<br>OXA -R	ACACAATACATATCAACTTCGC<br>AGTGTGTTTAGAATGGTGATC	813	55	Ramatla et al., 2022
	*bla*CARB	CARB-F<br>CARB-R	CAAGTACTTTYAAAACAATAGC<br>GCTGTAATACTCCKAGCAC	534	46	Jiang et al., 2013
	*bla*TEM	TEM-F<br>TEM-R	TTC TTG AAG ACG AAA GGG C<br>ACGCTCAGTGGAACGAAAAC	1150	55	Ramatla et al., 2022
	*bla*CTX-M-1	CTX-M-1-F<br>CTX-M-1-R	GTT ACA ATG TGT GAG AAG CAG<br>CCGTTTCCGCTATTACAAAC	1041	55	Liu et al., 2018
	*bla*CTX-M-2	CTX-M-2-F<br>CTX-M-2-R	ATGATGACTCAGAGCATTCGCCGC<br>TCAGAAACCGTGGGTTACGATTTT	876	55	Liu et al., 2018
	*bla*CTX-M-8	CTX-M-8-F<br>CTX-M-8-R	TGATGAGACATCGCGTTAAG<br>TAACCGTCGGTGACGATTTT	666	55	Gundran et al., 2019
	*bla*CTX-M-9	CTX-M-9-F<br>CTX-M-9-R	GTGACAAAGAGAGTGCAACGG<br>ATGATTCTCGCCGCTGAAGCC	856	55	Gundran et al., 2019
	*bla*CTX-M-15	CTX-M-15<br>CTX-M-15-R	CACACGTGGAATTTAGGGACT<br>GCCGTCTAAGGCGATAAACA	995	55	Liu et al., 2018
	*bla*CTX-M-25	CTX-M-25-F<br>CTX-M-25-R	GCACGATGACATTCGGG<br>AACCCACGATGTGGGTAGC	327	55	Gundran et al., 2019
**Carbapenems**						

	*bla*KPC	KPC-F<br>KPC-R	CGTCTAGTTCTGCTGTCTTG<br>CTTGTCATCCTTGTTAGGCG	789	52	Remya et al., 2018
	*bla*VIM	VIM-F<br>VIM-R	GGTCTCATTGTCCGTGATGGTG<br>GGAATCTCGCTCCCCTCTACCT	242	60	Sheikh et ai., 2014
**Aminoglycoside**						
	*str*A	strA-F<br>strA-R	CTTGGTGATAACGGCAATTC<br>CCAATCGCAGATAGAAGGC	548	55	Hong et al., 2018
	*str*B	strB-F<br>strB-R	ATCGTCAAGGGATTGAAACC<br>GGATCGTAGAACATATTGGC	509	56	Hong et al., 2018
	*aad*A	aadA-F<br>aadA-R	ATCCTTCGGCGCGATTTTG<br>GCAGCGCAATGACATTCTTG	283	56	Hong et al., 2018
	aadE	aadE-F<br>aadE-r	ATGGAATTATTCCCACCTGA<br>TCAAAACCCCTATTAAAGCC	386	50	Hong et al., 2018
	aac(6´)-Ib	aac(6´)-Ib-F<br>aac(6´)-Ib-R	TATGAGTGGCTAAATCGAT<br>CCCGCTTTCTCGTAGCA	395	55	Hong et al., 2018
	armA	armA-F<br>armA-R	CCGAAATGACAGTTCCTATC<br>GAAAATGAGTGCCTTGGAGG	846	56	
	*rmt*B	rmtB-F<br>rmtB-R	ATGAACATCAACGATGCCCT<br>CCTTCTGATTGGCTTATCCA	769	56	
**Phenicol**						
	*cat*I	catI-F<br>catI-R	GGTGATATGGGATAGTGTT<br>CCATCACATACTGCATGATG	349	60	Hong et al., 2018
	*cat*II	catII-F<br>catII-R	GATTGACCTGAATACCTGGAA<br>CCATCACATACTGCATGATG	567	60	Hong et al., 2018

	*cat*III	catIII-F<br>CatIII-R	CCATACTCATCCGATATTGA<br>CCATCACATACTGCATGATG	275	60	Hong et al., 2018
	*cat*IV	CatIV-F<br>catIV R	CCGGTAAAGCGAAATTGTAT<br>CCATCACATACTGCATGATG	451	60	Hong et al., 2018
	*flo*R	FloR-F<br>FloR-R	CGCCGTCATTCCTCACCTTC<br>GATCACGGGCCACGCTGTGTC	215	60	Hong et al., 2018

Supplementary References

1 Gundran RS, Cardenio PA, Villanueva MA, Sison FB, Benigno CC, Kreausukon K, Pichpol D, Punyapornwithaya V. Prevalence and distribution of *bla*CTX-M, *bla*SHV, blaTEM genes in extended-spectrum β-lactamase-producing *E. coli* isolates from broiler farms in the Philippines. BMC veterinary research. 2019;15:1-8.

2 Hong B, Ba Y, Niu L, Lou F, Zhang Z, Liu H, Pan Y, Zhao Y. A comprehensive research on antibiotic resistance genes in microbiota of aquatic animals. Frontiers in Microbiology. 2018;9:1617.

3 Jiang L, Hu X, Xu T, Zhang H, Sheng D, Yin D. Prevalence of antibiotic resistance genes and their relationship with antibiotics in the Huangpu River and the drinking water sources, Shanghai, China. Science of the Total Environment. 2013;458:267-72.

4 Liu G, Ding L, Han B, Piepers S, Naqvi SA, Barkema HW, Ali T, De Vliegher S, Xu S, Gao J. Characteristics of Escherichia coli isolated from bovine mastitis exposed to subminimum inhibitory concentrations of cefalotin or ceftazidime. BioMed Research International. 2018;2018.

5 Omolajaiye, S.A., Afolabi, K.O., Iweriebor, B.C. Pathotyping and antibiotic resistance profiling of *Escherichia coli* isolates from children with acute diarrhea in amatole district municipality of Eastern Cape, South Africa. *Biomed Res*. 18,1-10 (2020).

6 Ramatla T, Mileng K, Ndou R, Mphuti N, Syakalima M, Lekota KE, Thekisoe OM. Molecular detection of integrons, colistin and β-lactamase resistant genes in Salmonella enterica serovars enteritidis and typhimurium isolated from chickens and rats inhabiting poultry farms. Microorganisms. 2022;10(2):313.

7 Ramatla T, Mphuthi N, Gofaone K, Taioe MO, Thekisoe OM, Syakalima M. Identification of rodent species that infest poultry houses in Mafikeng, North West Province, South Africa. International Journal of Zoology. 2019;2019(1):1280578.

8 Ranjbar, R., Masoudimanesh, M., Dehkordi, F.S., Jonaidi-Jafari, N. and Rahimi, E., 2017. Shiga (Vero)-toxin producing Escherichia coli isolated from the hospital foods; virulence factors, o-serogroups and antimicrobial resistance properties. Antimicrobial Resistance & Infection Control, 6(1), p.4.

9 Remya, P., Shanthi, M., Sekar, U. Prevalence of blaKPC and its occurrence with other beta-lactamases in *Klebsiella pneumoniae*. *Journal of Laboratory Physicians*. 10(04),387-91 (2018).

10 Sheikh, F.A., Rostami, S., Jolodar, A., Tabatabaiefar, M.A., Khorvash, F., Saki, A., Shoja, S., Sheikhi, R. Detection of metallo-beta lactamases among carbapenem-resistant *Pseudomonas aeruginosa*. *Jundishapur journal of microbiology*. 7(11) (2014).

11 Sun W, Qian X, Gu J, Wang XJ, Duan ML. Mechanism and effect of temperature on variations in antibiotic resistance genes during anaerobic digestion of dairy manure. Scientific reports. 2016;6(1):30237.

12 Wolde, A., Deneke, Y., Sisay, T., Mathewos, M. Molecular Characterization and Antimicrobial Resistance of Pathogenic *Escherichia coli* Strains in Children from Wolaita Sodo, Southern Ethiopia. *J. Trop. Med*. 9166209 (2022).

13 Xu J, Xu Y, Wang H, Guo C, Qiu H, He Y, Zhang Y, Li X, Meng W. Occurrence of antibiotics and antibiotic resistance genes in a sewage treatment plant and its effluent-receiving river. Chemosphere. 2015;119:1379-85.
